# The Moderating Role of Anticipated Regret and Product Involvement on Online Impulsive Buying Behavior

**DOI:** 10.3389/fpsyg.2021.732459

**Published:** 2021-12-17

**Authors:** Bin Li, Minqi Hu, Xiaoxi Chen, Yongxin Lei

**Affiliations:** ^1^The Institute of Enterprise Development, Jinan University, Guangzhou, China; ^2^Management School, Jinan University, Guangzhou, China; ^3^Research Institute on Brand Innovation and Development of Guangzhou, Guangzhou, China

**Keywords:** anticipated regret, product involvement, online impulsive buying behavior, cognitive aspect, emotional aspect

## Abstract

Online impulsive buying behavior has drawn an increasing amount of attention from researchers and marketers as well; however, little research has explored how cognitive aspect and emotional aspect effect online impulsive buying together. The study examines the role of product involvement (cognitive aspect) and anticipated regret (emotional aspect) on the online impulsive buying behavior of the consumer. The results indicate that consumers who experienced downward anticipated regret showed more online impulsive buying behavior than those who experienced upward anticipated regret. Moreover, anticipated regret moderates the relationship between product involvement and online impulsive buying behavior, for participants who experienced downward anticipated regret showing more online impulsive buying behavior than those who experienced upward anticipated regret in the low product involvement group, but there is no differential between downward and upward anticipated regret in the high involvement product group. These findings suggest that anticipated regret helps consumers make more deliberative online shopping choices. The implications for both future research and online consumers are discussed.

## Introduction

People today enjoy convenient services provided by shopping websites. Reports from Internet Retailer (2019) indicated that Alibaba and Amazon jointly created a huge sales volume of $1.13 billion in 2018. During the COVID-19, the online store of Amazon achieved a 29% increase in sales (Davis, 2020). Online impulsive buying behavior makes a negative influence on consumers. People make their purchases online based on pictures and description from sellers. However, not all online information from sellers is believable. Consumers may experience negative affect due to online impulsive buying behavior (Ahn and Kahlor, 2020). Although consumers know how passive the situation is, they still engage in online impulsive buying behavior.

Online impulsive buying behavior is prevalent nowadays. Impulsive buying tendency urges consumers to buy the product immediately without hesitation (Chan et al., 2017). Research on impulsive buying behavior has concentrated their attention on external and internal factors. External factors help create atmosphere to urge the impulsive emotions, like shopping festival, quality of shopping website, and so on, of the consumers (Parboteeah et al., 2009; Liao et al., 2016; Guo et al., 2017; Hashmi et al., 2019; Ahn and Kahlor, 2020; Chen and Ku, 2021). Rather than being touched by arranged facilities and wrapped products, internal factors always relate to personal conditions and traits. Young consumers show higher impulsive buying tendency (Styvén et al., 2017). Online impulsive buying tendency is positively related to pressure (Moran and Kwak, 2015). When consumers feel time pressure, online impulsive buying behavior would become a carrier of their negative emotions (Sohn and Lee, 2017). More perceived relevance would promote the online impulsive buying tendency of the consumers (Dodoo and Wu, 2019). Under the situation of online shopping, it seems that people are more likely to shop without consideration of consequences.

It seems that emotion takes a leading position in online impulsive buying behavior. However, impulsive buying behavior also has its cognitive part. The cognitive function of emotion and the co-existence of cognition and emotion in the online shopping experience are supported as well (Nussbaum, 2003; Izogo and Jayawardhena, 2018). Product involvement links to the cognitive aspect of online impulsive buying behavior (Sohn and Lee, 2017). Danish Habib and Qayyum (2018) found the subsequence between cognitive aspect and emotional aspect in online impulsive buying behavior. This article agrees that cognition and emotion work mutually in online impulsive buying behavior. Emotion could link cognitive consequences, and reversely cognition could link emotional processes (Danish Habib and Qayyum, 2018; Chen et al., 2020; Chen and Ku, 2021).

Lesser studies press on affective aspects and cognitive aspects simultaneously. The decisions of online impulsive buying behavior depend on a combination of affective system and cognitive system. This study is conducted to shed more light on the mental process behind online impulsive buying behavior, especially how the cognitive aspect affects the emotional aspect. College students from China were invited to a simulated scenario to test product involvement, anticipated regret, and online impulsive buying behavior. The result was supposed to tell online impulsive buying behavior under the interaction of product involvement and anticipated regret.

## Hypothesis Development

### Theoretical Background

Easily accessible online shopping makes online impulsive buying closer to consumers. Impulsive buying behavior refers to the tendency of the consumers to buy spontaneously, unreflectively, immediately, and kinetically (Rook, 1987; Rook and Fisher, 1995). Features of impulsive buying behavior are the lack of information and insufficient evaluation of choices (Lim and Rashad, 2015; Xu et al., 2020). Control over self when faced with an online stimulus is also important in online impulsive buying behavior (Parboteeah et al., 2009). Most researchers use stimulus-organism-response theory and the theory of planned behavior to define impulsive buying behavior (Changa et al., 2011; Bilal Ahmad et al., 2019; Vazquez et al., 2020; Wu et al., 2020). Emotion state of mind is a significant mediator in stimulus-organism-response theory (Bilal Ahmad et al., 2019). Impulsiveness further facilitates the formation of unplanned impulsive buying behavior. To distinguish online impulsive buying from conventional online impulsive buying, Madhavaram and Laverie (2004) further defined online impulsive buying as the immediate reaction of consumers to external stimuli, especially stimuli of sensory information online stores.

In the extant literature, research has examined how website quality, review, social factor, and other factors influence online impulsive buying (Chang et al., 2012; Zhang and Zhang, 2015; Hashmi et al., 2019; Lin and Liu, 2019; Zhao et al., 2019). Product-related information could equip online consumers well when faced with potential risk on online shopping and post-purchase regret (Izah and Iskandar, 2019; Wu et al., 2020). Searching information could also help alleviate uncertain feeling in online shopping (Friedrich et al., 2019). Adequate information is indispensable for forming objective and effective evaluation. The lack of attention and evaluation brings much likelihood of online impulsive buying behavior (Drossos et al., 2014; Chan et al., 2017). Although cognitive resources and capacity of consumers are limited, they sometimes show a reluctant attitude to make more efforts on searching information for cognitive processes. This may involve specific classification of products.

Consumers are likely to let off their negative emotions by online shopping behavior. Researchers believed that impulsive buying behavior has become a form of emotional regulation (Fenton-O’Creevy et al., 2018; Sundström et al., 2019). When doing shopping, consumers choose different strategies that change from rational to affective (Peng et al., 2019). Time pressure under online shopping would influence the rational evaluation of consumers to low-involvement products (Zhao et al., 2019). Positive emotions like pleasure could increase purchase intention (Wakefield and Baker, 1998). Lin and Liu (2019) found that web pages could increase the online impulsive buying intention by color display since chromatic web page color displays lead to more aroused and stronger positive emotions. Writing reviews with emotional contents would increase impulsivity (Motyka et al., 2018). Negative emotions like regret also have an impact on online impulsive buying behavior. Izah and Iskandar (2019) proposed that the relationship between online impulsive buying and post-purchase regret is direct. During online shopping, impulsive consumers may also wonder necessity of this deal. No one can assure consumers the best time to buy something. Consumers may be afraid of possible regret from emotionally unplanned online shopping.

### Product Involvement and Online Impulsive Buying Behavior

Product involvement is a cognitive factor that affects the decision-making behavior of consumers. Zaichkowsky (1994) believed that involvement is the perceived relevance of an individual to internal needs and interests. High involvement means high product-personal relevance (Greenwald and Leavitt, 1984). Product involvement is subjective. Product value perceived by an individual, category of product, and correlation between an individual and a product affect the level of product involvement (Jones et al., 2003; Hong, 2015; Han and Kim, 2017). The subjective perception of consumers to products is crucial to product involvement. People under high product involvement would process information through the central route. People under low product involvement tend to process information through the peripheral route (Petty and Cacioppo, 1984). The affective part of product involvement presses on affective motive. The cognitive part of product involvement makes consumers focus on the utilitarian value of products (Drossos et al., 2014; Chan et al., 2017). Findings supported the relationship between high product involvement and positive emotional associations (Jaeger et al., 2018). High product involvement presses on the formation of affection and consumers would think before feeling. Low product involvement represents an affective need that influences cognition and consumers would feel before thinking (Belanche et al., 2017; Han and Kim, 2017; Verhagen and Bloemers, 2018). Low product involvement brings more online impulsive buying behavior, and high product involvement guides consumers to shop thoughtfully (Lloyd, 2014; Habib et al., 2021). Research could pay more attention to the relationship between product involvement and online impulsive buying behavior from an affective and rational perspective.

Product involvement affects the cognition and behavior of consumers. Product involvement contains affective and cognitive dimensions. The affective dimension of product involvement describes the feelings of consumers on the product. The cognitive dimension of product involvement describes information processing methods and the knowledge on products (Sandhe, 2020). Product involvement and cognitive ability have been proved to be related (Laaksonen, 1994; Marshall and Bell, 2004; Hong, 2015; Liu et al., 2020). High product involvement is accompanied by higher cognitive levels (Saqib et al., 2010). Impulsive buying is the cognitive response of consumers (Xiang et al., 2016; Kamboj et al., 2018; Vazquez et al., 2020). The cognitive dimension of product involvement and impulsiveness has a direct relationship with purchase intention (Drossos et al., 2014). Consumers will suffer from mistake shopping on high-involvement products since these products are important and expensive, but consequences from wrong shopping on low-involvement products are not unbearable (Jiang et al., 2015; Liu et al., 2020). When consumers are aware of the importance of goods, they will spend more time evaluating goods, and the possibility of online impulsive buying behavior thus decreases. Under low product involvement, the product is not highly relevant to consumers. Cognitive resources invested in collecting information will decrease accordingly. Consumers under low product involvement are more susceptible to marketing stimuli and are more likely to engage in online impulsive buying behavior.

**H1**. Product involvement has a significant and negative impact on online impulsive buying behavior, and participants do more online impulsive buying behavior when presented with low product involvement than with high product involvement.

### Anticipated Regret as a Moderator

Anticipated regret refers to the anxiety caused by the individual worrying about possible loss before making a decision, which can cause hesitation and doubt (Ritov and Baron, 1995). Counterfactual thinking before decision-making can lead to anticipated regrets. Conditional propositions like “what if” or “if only” are typical conceptualized expressions of counterfactual thoughts, which contain both an antecedent and a consequent (Roese, 1994). Directions of counterfactual thoughts tell the difference between alternatives and what happened. Counterfactual thoughts describe alternatives better than what happened, known as upward counterfactual thoughts; counterfactual thoughts describe what happened better than alternatives, known as downward counterfactual thoughts (Roese, 1994; Markman and McMullen, 2003; Epstude and Roese, 2008). Sandberg et al. (2016) explained anticipated regret stems from action regret for the commission of a behavior or inaction regret for the omission of a behavior. Participants who took advices felt more anticipated regret than participants who ignored them (Tzini and Jain, 2018).

Regret related more to cognitive consequence than merely reaction to stimuli (Zeelenberg and Pieters, 2007). Anticipated regret is a cognitive expectation about emotion and an emotionally inert (Robinson and Clore, 2002; Chun et al., 2019). Anticipated regret comes from personal assumption, but not the experience and reaction from anticipated regret is actually a virtual emotion (Chun et al., 2019). Research found that people under anticipated regret would make their decision more prudently (Hamilton et al., 2017; Verkijika, 2018; Ahn and Kahlor, 2020). Result from Hayashi et al. (2019) supported that anticipated regret is one of the antecedents of impulsive decision-making.

Emotion is an important antecedent that affects decision-making behavior. People tend to regret and they will try hard to prevent future regrets and avoid current regrets (Zeelenberg et al., 2006). People can use counterfactual thinking to anticipate the emotional consequences of imagined decision-making. Anticipated regrets in different directions will have different effects on the online impulsive buying behavior of consumers. When the direction of anticipated regret is upward, consumers believe that the price of the product will reduce and the current buying is a loss. People will abandon online impulsive buying behavior to avoid regret caused by the loss. When the direction of anticipated regret is downward, consumers believe that the future price of the product will be higher. They will feel regret if they miss the current price. At this time, the possibility of online impulsive buying behavior increases.

**H2**. The direction of anticipated regret has a significant and negative impact on online impulsive buying behavior, for participants who experienced downward anticipated regret showing more online impulsive buying behavior than those who experienced upward anticipated regret.

Anticipated regret relates to the cognitive process of online impulsive buying behavior. Anticipated regret not only assumes emotionally driven function but also conveys information to consumers and affects their cognitive style. This is not rejected by product involvement. The relationship between the chain of cognition and emotion and online impulsive buying behavior exists. Danish Habib and Qayyum (2018) found that low perceived risk and high perceived trust enhance the positive emotions of consumers when shopping online. Consumers with high positive emotions will spend more time browsing shopping websites. Online impulsive buying behavior will increase as a result. The model of Baumeister et al. (2007) supports the function of emotion to guide behavior through cognitive processes. In the formation process of online impulsive buying behavior, anticipated regret can directly drive emotions, and it can also act on the cognitive process to adjust the relationship between product involvement and online impulsive buying behavior.

**H3**. The direction of anticipated regret moderates the relationship between product involvement and online impulsive buying behavior.

## Methodology

### Sample

A total of 188 Chinese volunteers were recruited from a university in Wuhan city, China, and were randomly assigned to the four treatment groups. Researchers recruited volunteers, and bonus prizes were offered for participants. At first, participants were required to report their online shopping experience. This is the inclusion criterion on participants. Participants without online shopping experience and participants without complete response were excluded. The effective number of participants was 163 (46% male). The average age of the subjects was 21.07 + 2.07. This research also required participants to report basic information related to their online shopping experience.

In addition to the following measures, gender, age, length of experience on online shopping, and frequency of online shopping were controlled. Over 90% of participants reported their monthly income as less than 1,500 yuan. More than half of the participants had been shopping online for 1–3 years. Most participants would shop online 1–2 times a month. To better understand the mechanism of online impulsive buying behavior, we also included online shopping attitude and impulsive buying trait as control variables.

### Measures

Under the guidance of researchers, participants reported their basic information on online shopping experience first. After the manipulation test on product involvement, online shopping attitude and impulsive buying trait were tested. Then, participants were asked to finish the simulated scenario task. The whole process of the experiment was provided at an online platform called Wenjuanxing.

#### Simulated Scenario

The scenario task was revised from the former version (Rook and Fisher, 1995; Hetts et al., 2000). The revised simulated scenario task was based on shopping habit and actual expenditure of Chinese college students. At first, we designed different conditions for high product involvement (laptop) versus low product involvement (camera) based on the pretest. At the beginning of experiment, participants were invited to read the description on consumption decision of a college student. The product planned to buy online was a portable hard drive for school, and they found another product (camera vs. laptop) at a discount, which they yearn for a long time but didn’t plan to buy now. Product involvement was manipulated. Under high product involvement condition, the unplanned product was a laptop. Under low product involvement condition, the unplanned product was a camera. Under the situation of upward anticipated regret, participants were asked to imagine that the college student bought a laptop at discounted price and found the shopping website provided a lower price a week later. Participants were asked to think for a minute about how regret they were for buying the product. Under the situation of downward anticipated regret, participants were asked to imagine that the college student decided not to buy the camera and found it back to the original price. No other shopping websites provided lower price for the camera. After that, a chance of re-choosing was provided for participants. Participants were asked to think for a minute about how report for not buying the product and their intention on online impulsive buying.

#### Product Involvement

Personal Involvement Inventory (Zaichkowsky, 1994) was used to measure the product involvement of participants. The items of product involvement are as follows: important-unimportant, relevant-irrelevant, means nothing-means a lot to me, worthless-valuable, involving-uninvolving, and not needed-needed. Participants responded on a 7-point Likert scale with a higher score representing a higher level of product involvement. Cronbach’s α for pretest was 0.92.

Laptop and camera were chosen for manipulation on product involvement. An online pretest was conducted to measure the product involvement of laptop, cell phone, and camera, which were alternative material; 45 participants (55.6% male) were invited to an online pretest. Personal product inventory of Zaichkowsky (1994) was used. There was a significant difference among product involvement of laptop, cell phone, and camera, *F*(88, 2) = 37.56, *p* < 0.001, *SS* = 6,008.95, *MS* = 3,004.47, *M*_*mobile*_ = 54.40, *M*_*camera*_ = 44.58, *M*_*laptop*_ = 60.80. In pretest, the α for cell phone, camera, and laptop were 0.88, 0.97, and 0.92, respectively.

#### Impulsive Buying Trait

Impulsive Buying Trait Inventory (Zhang, 2010) was performed using the 7-point semantic differential scale. Twelve items were included in this scale, such as “When shopping, I like to buy it first and don’t care if I have enough money” and “As long as you like it, you should buy it immediately.” Items were scored on a 7-point Likert scale, ranging from 1 = *strongly disagree* to 7 = *strongly agree*. Cronbach’s α was 0.83.

#### Online Shopping Attitude

The online shopping attitude measure was revised by two pre-validated scales. This scale measured the perceived trust and risk on online shopping. The former three items were used to measure the perceived trust of consumers in online shopping, drawn from Han and Liu (2009), like “I think most shopping websites is trustworthy.” Items were scored on a 7-point Likert scale, ranging from 1 = *strongly disagree* to 7 = *strongly agree*. The latter four items were used to measure the perceived risk of consumers in online shopping, drawn from Li (2010), like “I think online shopping has product performance risks (fake, etc.).”Cronbach’s α was 0.75.

#### Online Impulsive Buying Behavior

Online impulsive buying behavior was measured by a single item. Participants were asked to re-choose after simulated scenario material. Under the situation of re-choosing, participants could decide whether to buy and which to buy.

## Results

### Descriptive Statistics and Correlations

The correlation matrix is reported in Table 1. Monthly income of participants (*p* < 0.05), impulsive buying trait (*p* < 0.01), and anticipated regret (*p* < 0.01) were significantly correlated with online impulsive buying behavior.

**TABLE 1:** The correlation matrix.

	Mean	SD	1	2	3	4	5	6	7	8	9	10
(1) Age	21.07	2.07	1									
(2) Gender	1.46	0.5	–0.015	1								
(3) Monthly income	1.58	0.74	–0.153	0.013	1							
(4) Length of experience on online shopping	2.56	0.85	0.219**	–0.107	0.128	1						
(5) Frequency of online shopping	1.39	0.64	–0.049	0.212**	–0.044	0.267**	1					
(6) Online shopping attitude	32.62	5.37	0.133	–0.056	0.076	0.1	–0.097	1				
(7) Impulsive buying trait	37.25	11.2	–0.142	0.07	0.278**	0.044	0.174*	–0.016	1			
(8) Product involvement	1	0.51	0.005	0.069	0.052	0.119	0.037	–0.006	0.142	1		
(9) Anticipated regret	1	0.5	0.059	0.031	0.112	0.01	0.025	0.055	–0.023	–0.019	1	
(10) Online impulsive buying behavior	2.81	1.28	–0.041	–0.103	0.156*	0.009	0.045	0.054	0.293**	–0.127	0.255**	1

*For gender, 1 = female, 2 = male. For monthly income, 1 = less than 1,000 yuan, 2 = 1,000–1,500 yuan, 3 = 1,500–2,000 yuan, 4 = more than 2,000 yuan. For length of experience on online shopping, 1 = 0–6 moths, 2 = 6–12 moths, 3 = 1–3 years, 4 = more than 3 years. For frequency of online shopping, 1 = 0–2 times, 2 = 2–5 times, 3 = 5–10 times, 4 = more than 10 times. For product involvement, 1 = low, 2 = high. For anticipated regret, 1 = upward, 2 = downward, 3 = controlled.*

*p < 0.05, two-tailed test, **p < 0.01, two-tailed test.

### Hypothesis Testing

#### Common Method Bias

A Harman single-factor test (Podsakoff and Organ, 1986) was conducted and found that the explained variance of the first principal component was 24.85%. Being below the cut-off value of 50% (Podsakoff et al., 2003), we concluded that common method bias was not a serious problem.

#### Manipulation Check on Product Category

Participants in the laptop condition reported higher involvement than those in the camera condition, *t*_(161)_ = –3.76, *p* < 0.0001, *M*_*camera*_ = 4.58, *M*_*laptop*_ = 5.32. The results confirmed that the manipulation of a product category was successful.

To test the hypothesis, we conducted a 2 (product involvement: high vs. low) × 2 (anticipated regret: upward vs. downward) ANOVA on online impulsive buying behavior while controlling for gender, age, and income. The analysis revealed that the main effect of product involvement on online impulsive buying behavior was significant, and participants with low product involvement (*M* = 2.98) showed more online impulsive buying behavior than participants with high product involvement (*M* = 2.65), *F*(1, 163) = 4.64, *p* < 0.05, η*^2^* = 0.03. Thus, H1 was supported.

The result also showed that the main effect of anticipated regret on online impulsive buying behavior was significant, and participants who experienced downward anticipated regret (*M* = 3.13) showed more online impulsive buying behavior than those experienced upward anticipated regret (*M* = 2.48), *F*(1, 163) = 12.57, *p* < 0.01, η*^2^* = 0.08. Therefore, H2 was supported.

**FIGURE 1:**
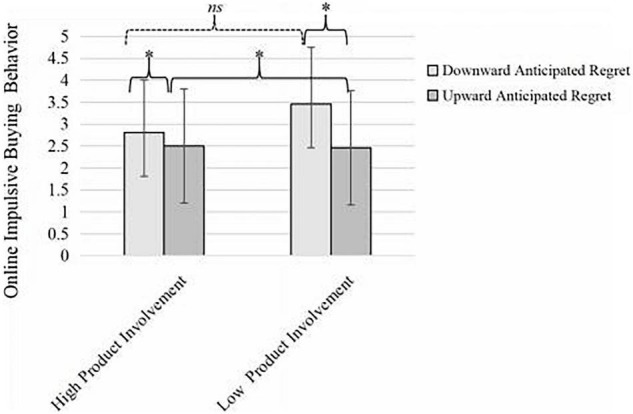
Moderating effect of anticipated regert. *p < 0.05. ns = Non-significant.

More important, the interaction effect between product involvement and anticipated regret on online impulsive buying behavior was significant, *F*(1, 163) = 4.11, *p* < 0.05, η*^2^* = 0.02. Under the condition of high product involvement, participants who experienced downward anticipated regret (*M* = 2.81) showed a non-significant difference in online impulsive buying behavior compared with participants who experienced upward anticipated regret (*M* = 2.50), *F*(1, 163) = 1.27, *df* = 1, *p* > 0.05. In the low product involvement condition, those who experienced downward anticipated regret (*M* = 3.46) acted significantly more online impulsive buying behavior than those who experienced upward anticipated regret (*M* = 2.46), *F* (1,163) = 13.25, *df* = 1, *p* < 0.01 (see Figure 1). Thus, H3 was supported.

## Discussion

This study investigated the interaction between anticipated regret and product involvement on online impulsive buying behavior. The main effect of product involvement and anticipated regret was supported. Anticipated regret significantly moderated the relationship between anticipated regret and product involvement.

Different product involvements have significantly different effects on online impulsive buying behavior. In the online shopping environment, product involvement affects online impulse shopping behaviors through the perception of consumers of products. Under the condition of higher product involvement, consumers are more willing to spend time and energy to collect information and evaluate products. While under the condition of lower product involvement, consumers lack motivation to engage information collection and product evaluation, and thus, their understanding of products is one-sided.

Consumers always pursue maximized utility in sales. In buying and selling transactions, merchants use product advantages to gain more, while consumers use product defects to pay less (Saqib et al., 2010). Online shopping magnifies this phenomenon. If consumers feel high perceived value, they will obtain much information about target products through the Internet. Thus, it is common that consumers abandon online impulsive buying behavior after receiving a bad evaluation on the product. Consumers with lower product involvement pay less cognitive effort to evaluate products. At this time, they may decide to shop immediately. Conclusions from this study provide supporting evidence for the relationship between product involvement and online impulsive buying behavior (Liang, 2012).

Product involvement reveals an emotional aspect in online impulsive buying behavior. Product involvement means engaged emotion (Gu et al., 2012). Kahneman (2011) asserted that emotion makes more impact on the decision than cognition. Low product involvement urges people make decision through affect heuristic, which means consumers would rely more on intuition and make decision more emotionally (Kahneman, 2011). When they do with low-involvement product, they spend less time or energy on searching information, and thus, they prefer to pay less for low-involvement product (Traylor, 1981; Ghasemaghaei and Hassanein, 2015). Similarly, consumers would take more factors into consideration for high involvement product (Stephen and Galak, 2012; Li, 2020). However, people may unexpectedly fail in online impulsive buying under low product involvement. People would persuade themselves to accept the shoddy product to avoid cognitive dissonance (Saqib et al., 2010). Time and energy people spend on information search largely might help mitigate potential risks they may suffer from. Objective and sufficient information is the basis of cognitive decision, which also influences the emotional aspect.

The relationship between anticipated regret directions and online impulsive buying behavior reflects regret aversion. When anticipated regret direction is downward, the current choice is better than the future plan. Consumers expect that choosing future plan will bring regret. To avoid future regret, consumers are more likely to choose current one and engage online impulsive buying behavior. The important influence of downward anticipated regret on online impulsive buying behavior has been supported. Positive expectations on future results can enlarge the possibility of online impulsive buying (Li et al., 2019). When the direction of anticipated regret is upward, the future choice is better than the current choice. Consumers under this situation are likely to choose future choice. In other words, upward anticipated regret makes it easier for consumers to give up online impulsive buying behavior.

This article is consistent with cognitive function of emotional factors. Results support the interaction between anticipated regret direction and product involvement. Previous studies have shown the combined effect of cognition and emotion on online impulsive buying behavior (Danish Habib and Qayyum, 2018; Fenton-O’Creevy et al., 2018). Choosing one from two always remind consumers of possibly consequent regret. Inaction inertia could help people avoid such regret (Tykocinski and Pittman, 1998; van Putten et al., 2013a,b).

The second alternative would remind people of the missed alternative. The fact on missed alternative causes people feel regret. Since anticipated regret exists, no matter how large attractiveness the second alternatives have, people still choose to omit it. People show reluctance to the second chance when they have missed the first chance in the same action domain, and this is inaction inertia (Tykocinski et al., 1995; Tykocinski and Ortmann, 2011). Researchers suggested that regret is an effective predictor of inaction inertia (Arkes et al., 2002; Sevdalis et al., 2006; van Putten et al., 2013b). Difference in attractiveness between two alternatives is an important condition of inaction inertia (Tykocinski et al., 2004; Zeelenberg et al., 2006). Just as Tykocinski et al. (1995) have proved in their experiment, the larger the difference in the attractiveness of the two chances exists, the larger the possibility of inaction inertia is.

Inaction inertia tends to happen in situations with anticipated regret (Butler and Highhouse, 2000; Tykocinski and Pittman, 2001). Upward anticipated regret in this research created a missed subsequent chance with larger attractiveness. In the initial situation, participants were assumed to have missed the chance to buy laptop or camera at a lower price. Upward anticipated regret comes from the counterfactual thinking on buying at discounted price or a better discounted price. When participants were asked to choose again, results show that they did not show clear preference on online impulsive buying. The reported indicator of online impulsive buying behavior is close to not buying; in other words, it is inaction. This inaction could be found in both high product involvement and low product involvement under upward anticipated regret. Missed subsequent attractive deals make people feel regret and keep inaction when faced with the second chance (van Putten et al., 2013a; Chen et al., 2021). Liu and Chou (2019) further discussed the performance of inaction inertia under different promotion strategies.

Downward anticipated regret comes from the comparison between missed alternative and inferior alternatives. In this study, participants did not take the first chance to have online impulsive buying on both camera and laptop. The situation under upward anticipated regret is that the latter alternative is better; condition under downward anticipated regret is that the current alternative is better (McConnell et al., 2000). Downward anticipated regret in this research comes from missed an attractive alternative. Participants under high product involvement are likely to take second alternative to have online impulsive buying behavior. The high product involvement means more cognitive effort and objective evaluation. Downward anticipated regret reminded participants of the current chance but evaluated potential risk stressed careful action (Dholakia, 2001; Sandhe, 2020). On the contrary, participants under low product involvement show a clear tend to have online impulsive buying behavior. Partially due to less engagement in efforts and spent time, consumers under low product involvement may care less about the risk of mispurchase (Kim, 2005). Trivial attributes could help mitigate inaction inertia in some extent, and products with low product involvement are probably considered a trivial product since people attach little importance to them (Kumar, 2019).

This study sheds light on existing literature on cognitive aspect and emotional aspect of online impulsive buying behavior. Discussion on cognition and emotion has been for decades (Gray, 1990; Clore and Palmer, 2009; Kahneman, 2011), and perspective combining emotion and cognition has been developed (Hasking et al., 2017; Raschle et al., 2017). This study provides more supporting evidence for the interaction between cognition and emotion. Product involvement involves activeness of cognitive resource, thus playing a cognitive part in online impulsive buying behavior (Sohn and Lee, 2017). Subsequence between cognitive aspect and emotional aspect in online impulsive buying behavior helps better understand the mental mechanism of consumers. Result from Danish Habib and Qayyum (2018) supported that the cognitive aspect could lead toward the emotion aspect in online impulsive buying behavior. Anticipated regret urges people to reconsider their decision rationally based on experience (Zeelenberg, 1999). Alternatively, cognition triggers emotional changes as well. Low product involvement is likely to stimulate impulsive emotions. People under high product involvement states tend to collect information actively, which can ease impulsive emotions. It can be seen that the two-way chain of cognition and emotion is particularly important in online impulsive buying behavior.

Second, the study highlighted the general application of regret theory. As negative emotion, people always try to avoid experiencing regret in their decision (Mourali et al., 2018). Online shopping is full of discount activities. Discounted products can easily trigger anticipated regret and a hotbed of inaction inertia (van Putten et al., 2013a; Chen et al., 2021). Inaction inertia helps better understand how online impulsive buying consumers react to product involvement and anticipated regret under irregular discounts. Moreover, based on the former research, this study further proposed the relationship between the direction of anticipated regret and inaction inertia, which enriches the current theoretical mechanism of inaction inertia (Sevdalis et al., 2006; Su et al., 2013).

Consumers should actively collect product information and take advantage of regret. Online sellers often use low-price gimmicks to attract consumers to focus entirely on the low prices of goods. Online impulsive buying behavior thus happens. Consumers are supposed to search more information about products and remind themselves of anticipated regret to mitigate impulsiveness. This can help reduce unnecessary online impulsive buying behavior. Since low involvement product is likely to attract the online impulsive buying behavior of the consumer, corporation related to such product should try to promote quality to reach higher consumer satisfaction. This would bring more returned customers to corporation. Meanwhile, government could provide technical guidance for corporations to provide low-involvement product. More supervision on corporation is needed for pushing more rational online shopping rather than impulsive online buying.

## Limitations and Future Research

The limitations of this study are as follows. First, this study only used scales and text descriptions to simulate online impulsive buying scenarios. The actual online shopping environment is different from this. Offering a simulation environment only by text description is an insufficient measure. Simulated materials used in this study were revised based on actual online shopping experience and consumption preference of the target sample. And the results also showed that no serious concern on common method bias. A deliberate behavioral lab could have helped this study receive better response, for example, a simulated shopping website. Future research should create more life-like simulation environment for better observing online impulsive buying behavior. Second, results based on student sample are limited. Student sample is one of the limitations of this study. Consumers engaged in online impulsive buying are available over all age groups and all professions. This study controlled age and monthly income to reduce unexpected influence. Future study should enlarge a range of sample rather than only focusing on specific group. Future research is supposed to use a larger sample source to expand the scope of application of the conclusions. Third, this study did not take income type into consideration. The privacy of online shopping can promote online impulsive buying behavior (Chih et al., 2012). However, the living expenses of Chinese college students mean “controlled” consumption, which represents the loss of privacy on online impulsive buying behavior. Future research could fully address the relationship between income types and online impulsive buying behavior.

## Conclusion

Regret always accompanies with decision in daily life. Anticipated regret helps consumers adjust current decision to avoid possible loss and future regret. This rule also works with different product types. This research is expected to help consumers understand the relationship between emotion and reason. Regret could buffer impulsive feelings in some extent. Consumers are supposed to establish healthy online shopping style by the take better advantage of their emotion.

## Data Availability Statement

The raw data supporting the conclusions of this article will be made available by the authors, without undue reservation.

## Ethics Statement

The studies involving human participants were reviewed and approved by Jinan University Management School Research Committee. The patients/participants provided their written informed consent to participate in this study.

## Author Contributions

BL and XC designed the study. XC and YL collected the data. BL and MH analyzed the data and draft the manuscript. MH, XC, and YL participated in the interpretation of the data. BL, MH, and XC revised the manuscript. All authors contributed to the article and approved the submitted version.

## Conflict of Interest

The authors declare that the research was conducted in the absence of any commercial or financial relationships that could be construed as a potential conflict of interest.

## Publisher’s Note

All claims expressed in this article are solely those of the authors and do not necessarily represent those of their affiliated organizations, or those of the publisher, the editors and the reviewers. Any product that may be evaluated in this article, or claim that may be made by its manufacturer, is not guaranteed or endorsed by the publisher.
