# Supplementary material for: The Moderating Role of Anticipated Regret and Product Involvement on Online Impulsive Buying Behavior
Source: Front Psychol. 2021 Dec 17;12:732459. doi: 10.3389/fpsyg.2021.732459 (PMC8719491; doi:10.3389/fpsyg.2021.732459)
Supplement: Supplementary file 1 [file Data_Sheet_1.docx]

**Materials of simulated scenario**

The following material was shown as an example of low product involvement.

Imagine that you are a 21-year-old college student with a monthly living allowance of 1,500 yuan. You have 400 yuan left to cover expense this month.7 days later you will get 1,500 yuan for next month. Further imagine that, you want a new digital camera and the money you save for it has reached 1,400 yuan yet. One day you plan to buy a mobile hard drive (about 150 yuan) online for schoolwork. After logging in a well-known shopping website you familiar with, you surprisingly discover that the camera you adored is on sale. The original price for the camera is 2,000 yuan, but it only costs 1,600 yuan after discount. A rechargeable battery, USB cable, CD-ROM, quick manual and warranty card are affiliated with the camera. The promotion lasts only for one day. The website guarantees for the good sold are authorized and licensed. They also declare that the camera is sold at the lowest price among all shopping websites. Goods with invoice will arrive within 7 days. The camera warranty is valid with corresponding invoice and nationwide warranty service is also available. The warranty period is one year.

Half of participants were asked to imagine the following scenario.

- 1. Group of upward anticipated regret

You decided to buy this camera after consideration but found that the website had offered a lower discount a week later. Think for a minute about how regret you were for buying this camera.

Further imagine that there is a chance to choose again, then which choice would you make? (1) I would buy only a mobile hard drive not a digital camera; (2) I would buy a mobile hard drive. I still want to buy a digital camera but do not; (3) I would decide not to buy mobile hard drive but advance my allowance for next month to buy a digital camera; (4) Advance my allowance for next month to buy a mobile hard drive and a digital camera at the same time; (5) Advance my allowance for next month to buy not only a mobile hard disk and a digital camera but also accessories related to digital cameras (such as original battery, 8G memory card, etc.).

The other half of participants were asked to imagine the following scenario.

- 1. Group of downward anticipated regret

You decided not to buy this camera after consideration. A week later, you found that the camera had backed to original price. The price for this camera is higher than 1,600 yuan in other shopping websites. Think for a minute about how regret you were for no buying this camera.

**Questionnaire**

*Scale Items for product involvement, online shopping attitude and impulsive buying trait*

| Construct | Measures |
| --- | --- |
| Product Involvement |  |
|  | Important |
|  | relevant |
|  | Valuable |
|  | Needed |
|  | Means a lot to me |
|  | Interesting |
|  | Appealing |
|  | Fascinating |
|  | Exciting |
|  | Involving |
| Online Shopping Attitude |  |
|  | I think most shopping websites are trustworthy. |
|  | I think most shopping websites are committed. |
|  | I think most shopping websites would pay close attention to consumers’ interests. |
|  | I think the risk of product performance (fake, etc.) exists when shopping online. |
|  | I think finance risk (credit card fraud online and loss of loan, etc.) exists when shopping online. |
|  | I think psychological risk (disclosure of personal information, etc.) exists when shopping online. |
|  | I think time-wasted risk (too much time spent on picking up goods and returning goods, etc.) exists when shopping online. |
| Impulsive Buying Trait |  |
|  | I would buy favored goods at once when shopping rather than worry about available money later. |
|  | Favored goods should be purchased immediately. |
|  | I believe that people should make a purchase as their pleases and exclude its usefulness. |
|  | Sometimes I shop for fun not for realistic needs. |
|  | I shop as planned usually. |
|  | I go shop without plan. |
|  | I am unaware of commodities’ practical function when pay for them. |
|  | I seldom give thoughts to my buying decision. |
|  | I would shop impulsively when at bad mood. |
|  | I would buy something to change my mood. |
|  | I do not want to take my eyes away from my favored goods. |
|  | I would feel unsatisfied if I can’t have my favored goods. |
